# Prediction of survival in oropharyngeal squamous cell carcinoma using machine learning algorithms: A study based on the surveillance, epidemiology, and end results database

**DOI:** 10.3389/fonc.2022.974678

**Published:** 2022-08-22

**Authors:** Su Il Kim, Jeong Wook Kang, Young-Gyu Eun, Young Chan Lee

**Affiliations:** Department of Otolaryngology-Head and Neck Surgery, Kyung Hee University School of Medicine, Seoul, South Korea

**Keywords:** oropharyngeal squamous cell carcinoma (OPSCC), overall survival (OS), conditional survival forest model, random survival forest model, DeepSurv model

## Abstract

**Background:**

We determined appropriate survival prediction machine learning models for patients with oropharyngeal squamous cell carcinoma (OPSCC) using the “Surveillance, Epidemiology, and End Results” (SEER) database.

**Methods:**

In total, 4039 patients diagnosed with OPSCC between 2004 and 2016 were enrolled in this study. In particular, 13 variables were selected and analyzed: age, sex, tumor grade, tumor size, neck dissection, radiation therapy, cancer directed surgery, chemotherapy, T stage, N stage, M stage, clinical stage, and human papillomavirus (HPV) status. The T-, N-, and clinical staging were reconstructed based on the American Joint Committee on Cancer (AJCC) Staging Manual, 8th Edition. The patients were randomly assigned to a development or test dataset at a 7:3 ratio. The extremely randomized survival tree (EST), conditional survival forest (CSF), and DeepSurv models were used to predict the overall and disease-specific survival in patients with OPSCC. A 10-fold cross-validation on a development dataset was used to build the training and internal validation data for all models. We evaluated the predictive performance of each model using test datasets.

**Results:**

A higher c-index value and lower integrated Brier score (IBS), root mean square error (RMSE), and mean absolute error (MAE) indicate a better performance from a machine learning model. The C-index was the highest for the DeepSurv model (0.77). The IBS was also the lowest in the DeepSurv model (0.08). However, the RMSE and RAE were the lowest for the CSF model.

**Conclusions:**

We demonstrated various machine-learning-based survival prediction models. The CSF model showed a better performance in predicting the survival of patients with OPSCC in terms of the RMSE and RAE. In this context, machine learning models based on personalized survival predictions can be used to stratify various complex risk factors. This could help in designing personalized treatments and predicting prognoses for patients.

## Introduction

Head and neck squamous cell carcinoma (HNSCC) is the sixth most common cancer worldwide, and includes all cancers occurring in the mucosa of the oral cavity, oropharynx, larynx, and hypopharynx (1). Unlike other HNSCCs that have shown declines in recent years, oropharyngeal squamous cell carcinoma (OPSCC) has shown a significant increase in incidence worldwide (2). This phenomenon is thought to be owing to an increase in the number of patients with human papillomavirus (HPV)-related OPSCC. A HPV-positive status in patients with OPSCC is associated with a better prognosis than an HPV-negative status (3). However, in addition to the HPV status in patients with OPSCC, other factors potentially affecting the patient prognosis should be considered.

Cox regression models have been used to evaluate the independent prognostic factors associated with the survival of patients with OPSCC using available clinical and pathological data. However, Cox regression models were not designed to predict an outcome, but rather to infer the impacts of variables on patient survival (4). Various machine learning methods have been designed to compensate for the limitations of the Cox progression models. Tree-based models are appealing owing to their logical and interpretable structures, as well as their ability to detect the complex interactions between covariates (5). Deep learning-based approaches are based on the automated learning of prognostic factors, without the need for prior assumptions on known factors (6). Thus, machine learning approaches may be considered as better approaches to predicting patient survival. However, it cannot be said which model is the most predictive of a prognosis; thus, they need to be analyzed and compared together.

In this study, we aimed to analyze the clinical data of patients with OPSCC using various machine learning models, aiming to determine the appropriate models for predicting survival in patients with OPSCC. We hypothesized that (1) investigation of a thousand OPSCC patients collected from the Surveillance, Epidemiology, and End Results (SEER) database would help to comprehensively consider the various variables, and (2) comparing the c-index, integrated Brier score (IBS), root mean square error (RMSE), and mean absolute error (MAE) in the machine learning models applied in this study would help in identifying the appropriate models.

## Materials and methods

### Patient cohort

The patient clinical data were obtained from the SEER program of the National Cancer Institute in the United States (https://seer.cancer.gov/, approval number:20922-Nov 2019). The inclusion criteria were as follows: (1) diagnosis of oropharyngeal cancer between 2004 and 2016, (2) tumor site in the oropharynx and/or tonsil, (3) histologic behavior of squamous cell carcinoma, and (4) patients had been tested for HPV status. Patients were excluded if they had missing data on survival months, cause of death, age, sex, tumor grade, tumor size, node status, or treatment methods (such as cancer-directed surgery, surgery, neck dissection, radiation therapy, or chemotherapy).

### Variable selection and reconfiguration

A total of 13 variables were included in this study: age, sex, tumor grade, tumor size, neck dissection, radiation therapy, cancer-directed surgery, chemotherapy, T stage, N stage, M stage, clinical stage, and HPV status. The age and tumor size were recorded using continuous methods (years and mm, respectively). The T-, N-, and clinical staging were reconstructed based on the American Joint Committee on Cancer (AJCC) Staging Manual, 8th Edition (7), and were categorized as “T0, TX, T1, T2, T3, T4, T4a, T4b,” “N0, NX, N1, N2, N2a, N2b, N2c, N3,” and “Stage I, II, III, IV, IVA, IVB, IVC,” respectively. These data were obtained using T- and N- and clinical staging according to the AJCC 7th Edition regarding the tumor size, regional node positive status, HPV status, and evaluation data provided by the SEER program. Whether neck dissections, radiation therapy, cancer-directed therapy, and chemotherapy were performed were also determined by referring to the SEER program.

### Applying multiple machine learning methods

**Figure 1:**
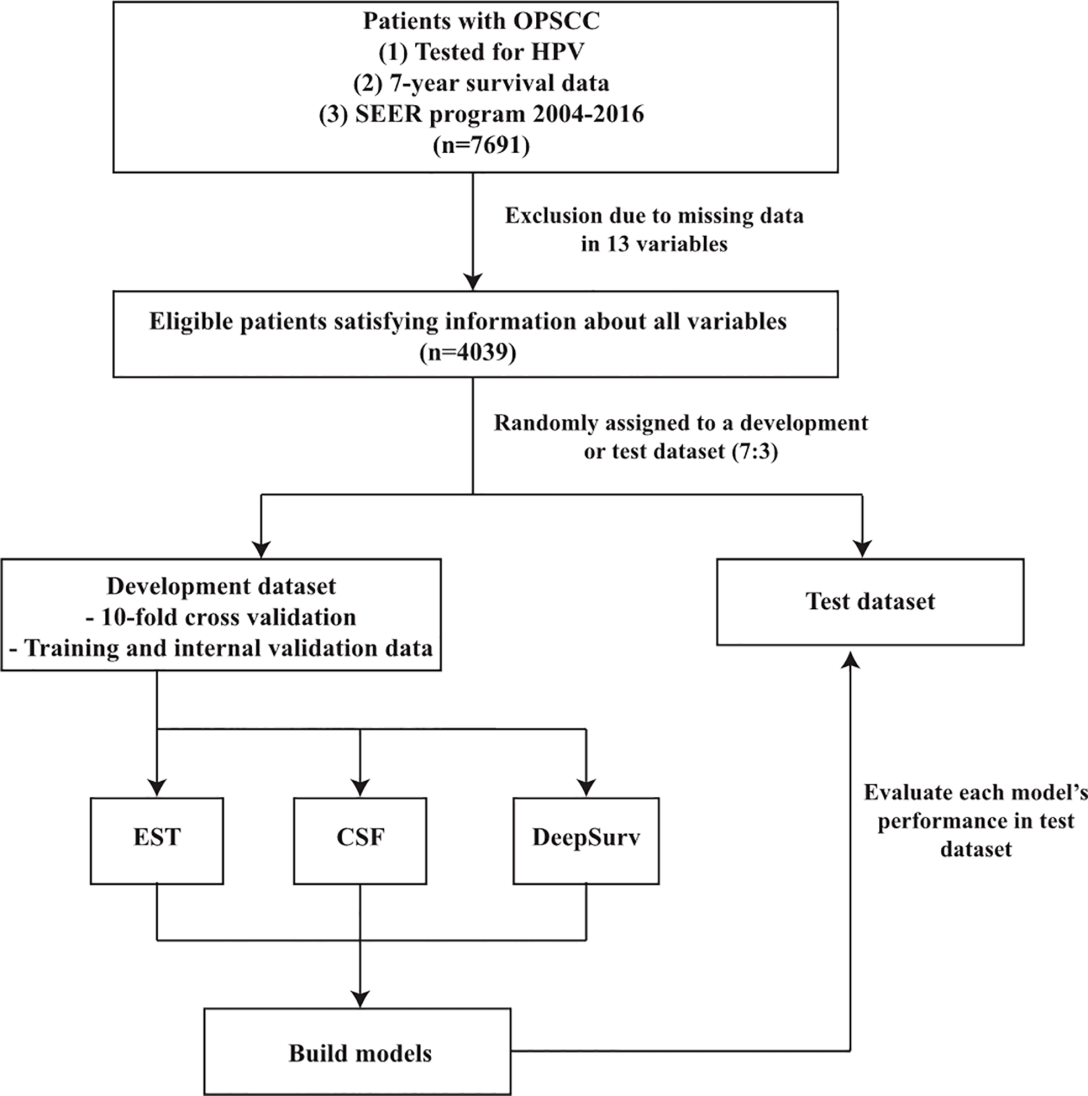
Flowchart of patient selection and study design. OPSCC, oropharyngeal squamous cell carcinoma; SEER, surveillance, epidemiology and end results; EST, Extremely randomized survival tree; CSF, Conditional survival forest.

The patients were randomly assigned to a development or test dataset in a 7:3 ratio. Three frequently and currently used machine learning methods, i.e., the extremely randomized survival tree (EST), conditional survival forest (CSF), and DeepSurv models were used to predict the overall and disease-specific survival in patients with OPSCC. A 10-fold cross-validation on a development dataset was used to build the training and internal validation data for all models. We evaluated the predictive performance of each model using the test datasets. The 13 variables specified above were used as the input data. The seven-year survival, cancer-specific death, and non-cancer-specific death data were used as the output data. **Figure 1** depicts the overall workflow of the patient selection and application of the machine learning methods.

### Evaluation of model performance

The predictive performances of the three machine learning models were evaluated using the C-index, IBS, RMSE, and MAE. A C-index close to or lower than 0.5 means that the performance of the model is no better than random outputs, and a higher C-index indicates a better performance of the model. IBSs are calculated using prediction error curves ranging from 0 (absolute performance) to 0.25 (8). The RMSE and MAE depict the differences between the actual and predicted prediction errors and survival values in each model. The RMSE gives extra weight to large errors, and the MAE gives equal weight to all errors. Smaller RMSE and MAE values indicate better performance (9).

All machine learning models were implemented in Python version 3.6 (Python Software Foundation for Statistical Computing, Wilmington, Delaware, USA) using PySurvival (10).

## Results

### Characteristics of the patients

First, the data of 7691 OPSCC patients who had been tested for HPV status were collected from the SEER program between 2004 and 2016. Moreover, 3652 patients were excluded because they were missing data for one or more of the 13 variables. Thus, 4039 OPSCC patients were analyzed using various machine learning methods (**Figure 1**).

**Table 1** describes the characteristics of the 4039 patients with OPSCC. In all patients, the age ranged from 0 to 94 years (mean =58.43 year), and 82.87% were male. The tumor size was described using mm units, and patients with microscopic focus or focus-only tumor size were assigned to the 1-mm tumor size. Approximately 34.96% of patients had undergone selective or radical neck dissection, regardless of chemotherapy and/or radiotherapy. Radiation therapy, cancer-directed surgery, and chemotherapy were performed for 88.19%, 61.43%, and 69.4% of the patients, respectively. The HPV-negative patients (25.06%) according to AJCC 8th Edition remained the same as those for the AJCC 7th Edition as listed in the SEER database, but the HPV-positive patients (74.94%) were modified in the new AJCC 8^th^ Edition data according to the AJCC 7th Edition and the tumor size, node size, and regional node evaluation section. The mean survival months were 34.72 months in the seven-year follow up.

**Table 1:** Clinical and pathological characteristics of patients with oropharyngeal squamous cell carcinoma (OPSCC).

Characteristics	N (total = 4039)
Age	
Mean [SD]	58.43 [9.65]
Median [min, max]	58 [0, 94]
Sex	
Male	3347 (82.87%)
Female	692 (17.13%)
Grade	
Well differentiated	155 (3.84%)
Moderately differentiated	1548 (38.33%)
Poorly differentiated	2281 (56.47%)
Undifferentiated/anaplastic	55 (1.36%)
Tumor size (mm)	
Mean [SD]	29.66 [18.52]
Median [min, max]	28.00 [0.00, 510.00]
Neck dissection	
Selective or radical neck dissection	1412 (34.96%)
Sentinel or regional node biopsy	557 (13.79%)
No	2070 (51.25%)
Radiation therapy	
Yes	3562 (88.19%)
No	477 (11.81%)
Cancer directed surgery	
Yes	2481 (61.43%)
No	1558 (38.57%)
Chemotherapy	
Yes	2803 (69.4%)
No	1236 (30.6%)
T stage (American Joint Committee on Cancer (AJCC) 8th edition)	
T0	7 (0.17%)
TX	19 (0.47%)
T1	1197 (29.64%)
T2	1689 (41.82%)
T3	646 (15.99%)
T4	299 (7.40%)
T4a	119 (2.95%)
T4b	63 (1.56%)
N stage (AJCC 8th edition)	
N0	667 (16.51%)
NX	9 (0.22%)
N1	2378 (58.88%)
N2	295 (7.30%)
N2a	103 (2.55%)
N2b	309 (7.65%)
N2c	104 (2.57%)
N3	174 (4.31%)
M stage (AJCC 8th edition)	
M0	3932 (97.35%)
M1	107 (2.65%)
Clinical stage (AJCC 8th edition)	
Stage I	2101 (52.02%)
Stage II	631 (15.62%)
Stage III	593 (14.68%)
Stage IV	62 (1.54%)
Stage IVA	506 (12.53%)
Stage IVB	101 (2.50%)
Stage IVC	45 (1.11%)
HPV status	
Positive	3027 (74.94%)
Negative	1012 (25.06%)
Survival months	
Mean [SD]	34.72 [20.77]
Median [min, max]	32.00 [0.00, 83.00]
Cause of death; cancer specific	
Death	515 (12.75%)
Alive	3524 (87.25%)
Cause of death; non-cancer specific	
Death	779 (19.29%)
Alive	3260 (80.71%)

SD, standard deviation.

### Application and analysis of each model

**Figure 2:**
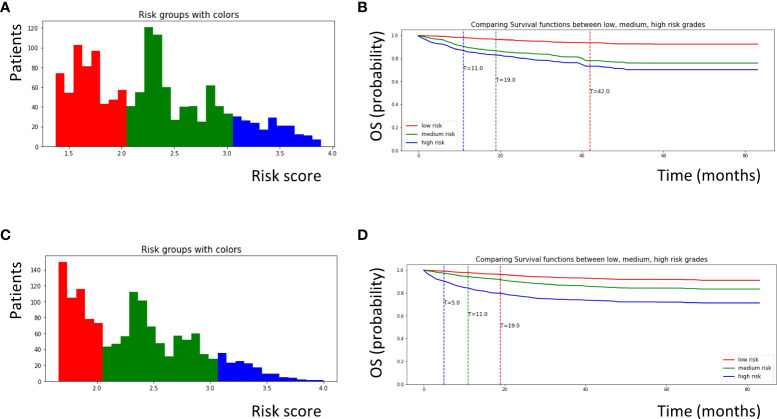
Survival curves for patients with OPSCC classified according to risk scores calculated by each machine learning model. **(A)** Patients with OPSCC were classified into low, medium, and high risk groups according to risk scores calculated by the EST model. Patients with a score of 0–2.0 were defined as low risk (red color), patients with a score of 2.0–3.0 were defined as medium risk (green color), and patients with a score of 3.0–4.0 were defined as high risk (blue color). **(B)** Survival curves for three patients who was randomly selected from each group obtained from the EST model. **(C)** Similarly, patients with OPSCC were classified into three groups according to risk scores calculated by the CSF model. Patients with a score of 0–2.0 were defined as low risk (red color), patients with a score of 2.0–3.0 were defined as medium risk (green color), and patients with a score of 3.0-4.0 were defined as high risk (blue color). **(D)** Survival curves for three patients randomly selected from each group obtained from the CSF model. OPSCC, oropharyngeal squamous cell carcinoma; EST, Extremely randomized survival tree; CSF, Conditional survival forest.

In the EST model, we used 20 minimum node sizes, 200 trees, and 1000 random splits. The risk factors for OPSCC were calculated using the EST model. The risk scores ranged from 0 to 4, and patients were categorized into three groups (low-, medium-, and high-risk) according to these scores (**Figure 2A**). One patient was randomly selected from each group for our new risk-related staging system, and survival curves for 72 months are depicted for these three patients (**Figure 2B**). We further analyzed the importance of each variable (**Table 2**), and cancer-directed surgery showed the highest importance (13.88), followed by HPV status (11.11), radiation therapy (9.06), T stage (8.41), and M stage (8.35).

**Table 2:** Importance of various variables in each machine learning model.

	EST		CSF	
Variable (in order of importance)	Feature	Importance	Feature	Importance
1	Cancer directed surgery	13.88	Cancer directed surgery	11.53
2	HPV	11.11	Tumor size	8.80
3	Radiation therapy	9.06	HPV	8.74
4	T stage	8.41	Radiation therapy	7.82
5	M stage	8.35	Age	7.08

EST, Extremely randomized survival tree; CSF, Conditional survival forest.

In the CSF model, we used 20 minimum node sizes, 200 trees, and a 0.05 alpha. Similar to the EST model, the risk factors for the patients were calculated using the CSF model. The risk scores ranged from 0 to 4, and the patients were categorized into three groups (**Figure 2C**). Using the same methods, survival curves were obtained for the three patients (**Figure 2D**). Cancer-directed surgery showed the highest importance (11.53), similar to the EST model; however, the tumor size (8.80) and age (7.08) also showed high importance compared to the EST model.

In the DeepSurv model, we used xav_uniform as the initial method and an adaptive moment estimation optimizer with a learning rate of 0.001 for the neural network, with one hidden layer activation function = “BentiIdentity” (for the input-hidden layer) and node size = 150 (11). Dropout, batch normalization, and L1 and L2 regularization were performed during the training.

### Comparison of each model performance

**Figure 3:**
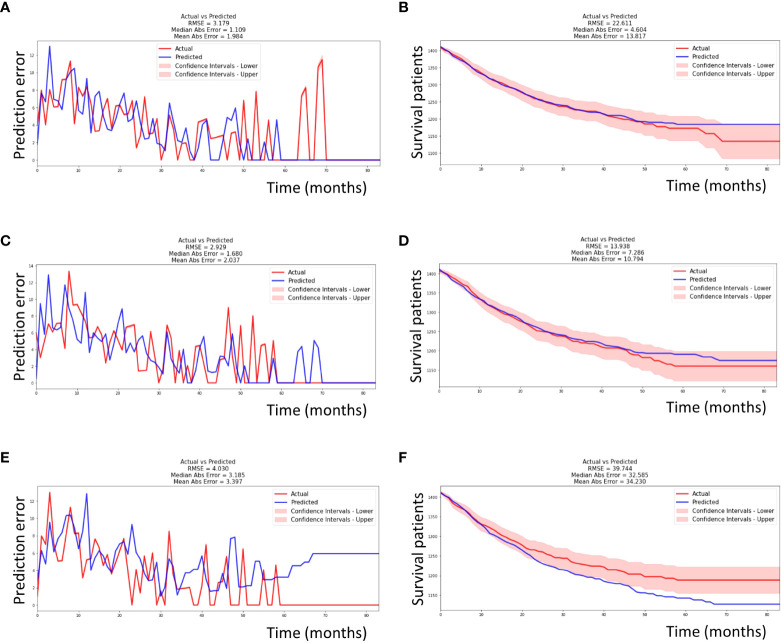
Performance of each machine learning model. **(A)** The EST model had an RMSE of 3.719 and MAE of 1.984 during seven-years of follow-up time in testing set. **(B)** The EST model had an RMSE of 22.611 and MAE of 13.817 in survival curve prediction. **(C)** Similarly, the CSF model had an RMSE of 2.929 and MAE of 2.037. **(D)** The CSF model had an RMSE of 13.938 and MAE of 10.794 in survival curve prediction. **(E)** Similarly, the DeepSurv model had an RMSE of 4.030 and MAE of 3.397. **(F)** The DeepSurv model had an RMSE of 39.744 and MAE of 34.230 in survival curve prediction. The actual and predicted survival curves were within the confidence intervals in only the CSF model. EST, Extremely randomized survival tree; CSF, Conditional survival forest; RMSE, root mean square error; MAE, mean absolute error.

As mentioned above, higher values of the C-index and lower values of the IBS, RMSE, and MAE indicate a better performance of a machine learning model. The C-index was the highest in the DeepSurv model (0.77), followed by the EST and CSF models (0.76 and 0.72, respectively). The IBS was the lowest in DeepSurv model (0.08), followed by the EST and CSF models (0.10 and 0.10, respectively). However, the RMSE and RAE (depicting the differences between the actual and predicted survival values) were the lowest in the CSF model (13.398 and 10.794, respectively), followed by the EST (22.611 and 13.817, respectively) and DeepSurv models (39.744 and 34.230, respectively) (**Table 3** and **Figure 3**).

**Table 3:** Comparison of each machine learning model’s performance.

	C-index	IBS	RMSE in prediction error	MAE in prediction error	RMSE in survival values	MAE in survival values
EST	0.76	0.10	3.179	1.984	22.611	13.817
CSF	0.72	0.10	2.929	2.037	13.938	10.794
DeepSurv	0.77	0.08	4.030	3.397	39.744	34.230

IBS, integrated Brier score; RMSE, root mean square error; MAE, mean absolute error; EST, extremely randomized survival tree; CSF, conditional survival forest.

## Discussion

The therapeutic methods for early-stage OPSCC are surgery or radiation therapy, and appropriate methods are commonly selected according to the patient and clinician preferences and conditions (12). Parsons et al. found through a meta-analysis that patients with OPSCC experienced similar overall survival rates regardless of whether they were treated with surgery or definitive radio(chemo)therapy (13). In the case of locally advanced OPSCC according to the National Cancer Database, primary surgery with radio(chemo)therapy showed improved survival compared to primary radiation-based treatment (14). In addition, patients with HPV-negative OPSCC had a significantly worse prognosis than those with HPV-positive OPSCC (15). In addition, other factors, including age, sex, and T/N/M staging also affected the prognosis of patients with OPSCC. Thus, we aimed to predict the prognoses of patients with OPSCC by comprehensively analyzing various prognostic factors using frequently used machine-learning models.

In this study, we used the SEER database because it provides large population-based information that cannot be obtained from just one medical center. The SEER cohort provided staging information based on the AJCC 7^TH^ Edition, which does not reflect a new tumor grading system according to HPV status. Other studies showing the factors influencing the prognosis of OPSCC patients also used TNM staging based on the AJCC 7^th^ Edition. However, we assumed that reconstructing the staging system to suit the new AJCC 8^th^ Edition would better demonstrate their impacts on prognosis in OPSCC when considering HPV status. In addition, clinical data such as age and sex, detailed tumor size, grade, and applied therapeutic modalities (chemotherapy, neck dissection, radiotherapy, and cancer-directed surgery) were obtained and analyzed comprehensively.

Until recently, conventional Cox regression models were mostly used to predict the prognoses of OPSCC patients (16, 17). However, two effects might be simplified or overlooked in the process of interpreting Cox regression models: “Effect modification,” i.e., the causal effect of one exposure within the strata of another exposure of interest and “Interaction,” i.e., the causal effect of two exposures together in an area of interest (18). In addition, the Cox regression model cannot recognize the complex nonlinear relationships between variables. Statistically reinforced machine learning approaches would be beneficial for compensating for problems such as higher-order interactions, context dependencies, nonlinearity, and variable interactions (19). Thus, we analyzed and compared three frequently used machine learning methods –EST, CSF, and DeepSurv– to consider nonlinearity and reduce the interactions and effect modifications in the variables of the SEER cohort.

Interestingly, cancer-directed surgery was the most important variable for predicting the prognosis of patients with OPSCC in both the EST and CSF models. HPV status and radiation therapy were also important variables in both the EST and CSF models. These results suggest that selection of an appropriate therapeutic modality, including surgery and radiotherapy, might be more important in predicting the prognosis of patients with OPSCC than the TNM staging alone. In addition, we evaluated the performance of each machine learning method using variable scales: the C-index, IBS, RMSE, and MAE. The C-index and IBS results indicated that the DeepSurv model showed better performance in predicting the survival of patients with HNSCC. Hao et al. also found that the DeepSurv model performed better than other machine learning models in terms of the C-index, IBS, and area under the curve for survival prediction performance (20). The DeepSurv model uses a feed-forward network to learn the relationships between the covariates and hazard function, thus capturing the time-dependent influences of the covariates on survival (21).

However, in terms of the RMSE and RAE, the CSF model performed better than the EST and DeepSurv models. The EST includes a random survival forest, uses equal weights on all terminal nodes, and analyzes time-to-event data comprising covariates with many split-points. The CSF model was superior in analyzing time-to-event data that consisted of covariates with fewer split points (22). Deep learning models have exhibited unprecedented performance in quite a few applications in academia and industry, but there are many disadvantages, such as their complex geometric transformations, which often require big data (23). Thus, it might be important to analyze the various survival models together so that they can be used to compensate for each other.

Our study had the following limitations. First, the SEER database showed limited information; for example, it did not include data regarding smoking and alcohol histories, or recurrence after treatment. Above all, the SEER database did not include information about detailed chemotherapy methods and extranodal extension, which are important prognostic factor of OPSCC. To compensate limited information, we analyzed all available treatment methods such as neck dissection, radiation, cancer directed surgery and chemotherapy together. Second, we reconstructed the AJCC 8^th^ Edition based on the AJCC 7th Edition, tumor size, regional node, and HPV status. The AJCC 8th Edition requires the HPV status; thus, we had to exclude OPSCC patients without HPV status data. However, we were able to analyze in more detail the effect of the AJCC 8^th^ Edition on the prognosis of OPSCC. Third, this study included patients treated from 2004 to 2016. Advances in surgical technology might influence the prognoses of patients; however, detailed information on such surgical methods was not available.

Nevertheless, to the best of our knowledge, this is the first study to analyze the prognosis of OPSCC patients using various machine learning methods based on the AJCC 8th Edition. We found that therapeutic methods, such as surgical therapy and radiotherapy, had a more important effect on prognosis than T/N/M staging alone. In addition, we found that various machine learning models showed different important factors affecting the prognosis. Further studies including more detailed clinical and pathological data will help improve the accuracy of survival prediction using the various machine learning models.

In conclusion, we demonstrated that the survival predictions from various machine-learning models (EST, CSF, and DeepSurv) are feasible and accurate. In particular, the CSF model showed a better performance in predicting the survival of patients with OPSCC in terms of the RMSE and RAE. Machine learning models based on personalized survival prediction can also be used to stratify various complex risk factors. This will help in designing personalized treatments and predicting the prognoses of patients.

## Data availability statement

Publicly available datasets were analyzed in this study. This data can be found here: https://seer.cancer.gov/.

## Author Contributions

SK: study design, data collection and analysis, writing, revising the article, and final approval of the version; JK: data collection and analysis, revising article, and final approval of the version; Y-GE: data collection and analysis, revising article, and final approval of the version; YL: study design, data collection and analysis, revising article, final approval of the version, and supervision of the study. All authors contributed to the article and approved the submitted version.

## Funding

This study was supported by a National Research Foundation of Korea (NRF) grant funded by the Korean government (MSIT) (No. 2020R1F1A1069338). This work was also supported by the National Research Foundation of Korea Grant funded by the Korean government, Ministry of Science, and ICT (grant no.2021M3A9E8015437).

## Acknowledgments

We would like to thank Editage (www.editage.co.kr) for English language editing.

## Conflict of interest

The authors declare that the research was conducted in the absence of any commercial or financial relationships that could be construed as a potential conflict of interest.

## Publisher’s note

All claims expressed in this article are solely those of the authors and do not necessarily represent those of their affiliated organizations, or those of the publisher, the editors and the reviewers. Any product that may be evaluated in this article, or claim that may be made by its manufacturer, is not guaranteed or endorsed by the publisher.

## References

[1] Kamangar F Dores GM Anderson WF. Patterns of cancer incidence, mortality, and prevalence across five continents: defining priorities to reduce cancer disparities in different geographic regions of the world. J Clin Oncol (2006) 24:2137–50. doi: 10.1200/JCO.2005.05.2308 16682732

[2] Rainsbury JW Ahmed W Williams HK Roberts S Paleri V Mehanna H. Prognostic biomarkers of survival in oropharyngeal squamous cell carcinoma: systematic review and meta-analysis. Head Neck (2013) 35:1048–55. doi: 10.1002/hed.22950 22997051

[3] Ragin CC Taioli E. Survival of squamous cell carcinoma of the head and neck in relation to human papillomavirus infection: review and meta-analysis. Int J Cancer (2007) 121:1813–20. doi: 10.1002/ijc.22851 17546592

[4] Kim DW Lee S Kwon S Nam W Cha IH Kim HJ. Deep learning-based survival prediction of oral cancer patients. Sci Rep (2019) 9:6994. doi: 10.1038/s41598-019-43372-7 31061433 PMC6502856

[5] Bertsimas D Dunn J Gibson E Orfanoudaki A. Optimal survival trees. Mach Learn (2022) 1–73. doi: 10.1007/s10994-021-06117-0

[6] Wulczyn E Steiner DF Xu Z Sadhwani A Wang H Flament-Auvigne I. Deep learning-based survival prediction for multiple cancer types using histopathology images. PloS One (2020) 15:e0233678. doi: 10.1371/journal.pone.0233678 32555646 PMC7299324

[7] Zanoni DK Patel SG Shah JP. Changes in the 8th edition of the american joint committee on cancer (ajcc) staging of head and neck cancer: rationale and implications. Curr Oncol Rep (2019) 21:52. doi: 10.1007/s11912-019-0799-x 30997577 PMC6528815

[8] Lawless JF Yuan Y. Estimation of prediction error for survival models. Stat Med (2010) 29:262–74. doi: 10.1002/sim.3758 19882678

[9] Erdman EA Young LD Bernson DL Bauer C Chui K Stopka TJ. A novel imputation approach for sharing protected public health data. Am J Public Health (2021) 111:1830–8. doi: 10.2105/AJPH.2021.306432 PMC8561211 34529494

[10] Fotso S. PySurvival: open source package for survival analysis modeling (2019). Available at: https://square.github.io/pysurvival/ (Accessed February 16, 2022).

[11] Kingma DP Ba JL Adam A. (2015). A method for stochastic optimization, in: nternational Conference on Learning Representations (ICLR)(2015), San Diego, CA.

[12] Hilal L Moukarbel R Ollaik F Yang P Youssef B. Patient selection for surgery vs radiotherapy for early stage oropharyngeal cancer. Cancer Control (2021) 28:10732748211050770. doi: 10.1177/10732748211050770 34936505 PMC8704187

[13] Parsons JT Mendenhall WM Stringer SP Amdur RJ Hinerman RW Villaret DB. Squamous cell carcinoma of the oropharynx: surgery, radiation therapy, or both. Cancer (2002) 94:2967–80. doi: 10.1002/cncr.10567 12115386

[14] Kamran SC Qureshi MM Jalisi S Salama A Grillone G Truong MT. Primary surgery versus primary radiation-based treatment for locally advanced oropharyngeal cancer. Laryngoscope (2018) 128:1353–64. doi: 10.1002/lary.26903 28988426

[15] Fakhry C Westra WH Li S Cmelak A Ridge JA Pinto H. Improved survival of patients with human papillomavirus-positive head and neck squamous cell carcinoma in a prospective clinical trial. J Natl Cancer Inst (2008) 100:261–9. doi: 10.1093/jnci/djn011 18270337

[16] Pagedar NA Chioreso C Schlichting JA Lynch CF Charlton ME. Treatment selection in oropharyngeal cancer: a surveillance, epidemiology, and end results (SEER) patterns of care analysis. Cancer Causes Control (2017) 28:1085–93. doi: 10.1007/s10552-017-0938-3 PMC5771802 28815336

[17] Kwak MS Eun YG Lee YC. Benefit of postoperative radiotherapy in patients with oropharyngeal squamous cell carcinoma in human papillomavirus (HPV) era: A surveillance, epidemiology, and end results (SEER) database analysis. Surgery (2021) 170:541–9. doi: 10.1016/j.surg.2021.01.034 33663877

[18] Knol MJ VanderWeele TJ. Recommendations for presenting analyses of effect modification and interaction. Int J Epidemiol (2012) 41:514–20. doi: 10.1093/ije/dyr218 PMC3324457 22253321

[19] Ryo M Rillig MC. Statistically reinforced machine learning for nonlinear patterns and variable interactions. Ecosphere (2017) 8:e01976. doi: 10.1002/ecs2.1976

[20] Hao L Kim J Kwon S Ha ID. Deep learning-based survival analysis for high-dimensional survival data. Mathematics (2021) 9:1244. doi: 10.3390/math9111244

[21] Lee C Yoon J Schaar MV. Dynamic-DeepHit: A deep learning approach for dynamic survival analysis with competing risks based on longitudinal data. IEEE Trans Bio Med Eng (2020) 67:122–33. doi: 10.1109/TBME.2019.2909027 30951460

[22] Nasejje JB Mwambi H Dheda K Lesosky M. A comparison of the conditional inference survival forest model to random survival forests based on a simulation study as well as on two applications with time-to-event data. BMC Med Res Methodol (2017) 17:115. doi: 10.1186/s12874-017-0383-8 28754093 PMC5534080

[23] Tang B Pan Z Yin K Khateeb A. Recent advances of deep learning in bioinformatics and computational biology. Front Genet (2019) 10:214. doi: 10.3389/fgene.2019.00214 30972100 PMC6443823

